# Identifying Synergistic Mechanisms of Community-Led Policy, Systems, and Environmental Change for Childhood Obesity Prevention in the Multi-Site Catalyzing Communities Initiative

**DOI:** 10.1007/s11524-025-01046-y

**Published:** 2026-01-07

**Authors:** Travis R. Moore, Yuilyn A. Chang Chusan, Rebecca Neergaard, Erin Hennessy, Larissa Calancie, Christina D. Economos, Shiriki Kumanyika

**Affiliations:** 1 Friedman School of Nutrition Science and Policy, Tufts University, 150 Harrison Ave, Boston, MA 0211 USA; 2 School of Public Health, Boston University, 715 Albany Streett, Boston, MA 02118 USA; 3 Division of Biostatistics, Epidemiology, and Informatics, Perelman School of Medicine, University of Pennsylvania, 423 Guardian Drive, Philadelphia, PA 19104 USA

**Keywords:** Catalyzing Communities, Policy, Systems, and Environmental Change, Getting to Equity Framework, Implementation, Childhood obesity, Prevention, Community development, Community engagement

## Abstract

This evaluation reports how the Catalyzing Communities Initiative, a whole-of-community, multi-site intervention, supported equity-focused policy, systems, and environmental (PSE) changes to prevent childhood obesity in three U.S. communities from 2023 to 2024. We conducted a mixed-methods evaluation using survey data from 35 committee members (multisector representatives selected by local health-promotion leaders) and interviews with 29 committee members across three communities. We used a three-framework analytic approach: the Getting to Equity Framework was used to classify PSE changes, the Consolidated Framework for Implementation Research guided analysis of contextual influences, and Ripple Effects Mapping, a systems science method, identified how PSE-related actions created chains of perceived impacts over time. Committee members reported a total of 339 PSE impacts, defined as perceived changes attributed to specific PSE-related actions, such as shifts in partnerships, practices, or community conditions. The majority of impacts fell within Getting to Equity Quadrants 4 (increasing community capacity and opportunity) and 1 (increasing healthy options). Systems thinking tools that were used by committee members as part of the Catalyzing Communities Initiative were noted to enhance cross-sector coordination. Ripple effect maps revealed reinforcing loops, such as systems-informed community action and community mobilization, that enhanced cross-sector collaboration and contributed to synergistic change. Implementation facilitators of change included strong leadership and supportive policy environments, while key barriers included affordability, stigma, and exposure to community violence. Findings illustrate how integrating equity frameworks, implementation science, and Ripple Effects Mapping can reveal mechanisms through which local actions promote equity-oriented change.

**Supplementary Information:**

The online version contains supplementary material available at 10.1007/s11524-025-01046-y.

## Introduction

Whole-of-community approaches to addressing childhood obesity and related health inequities have gained prominence in communities in the United States (U.S.) and elsewhere [1]. This reflects an emerging understanding that childhood obesity is a complex problem influenced by numerous sectors operating at multiple ecological levels, and that reproduces broad inequities that affect marginalized populations [2–5]. The policy, systems, and environmental (PSE) changes needed to oppose or mitigate these drivers require equity-focused, context-specific, and community-driven interventions that are comprehensive in nature and preferably guided by systems thinking that considers interdependencies and possible synergies among the various components [5, 6].

The Catalyzing Communities Initiative (CCI) was designed to implement and evaluate a community-driven approach in which community partners and research teams build meaningful partnerships to support the mobilization of multisector community leadership to improve child health equity, typically focusing on nutrition, physical activity, and healthy weight [3, 7–10]. Grounded in community-based participatory research and systems science, CCI is guided by the Stakeholder-Driven Community Diffusion (SDCD) theory [7, 11] which posits that engaging influential community stakeholders in facilitated, collaborative processes can catalyze the diffusion of systems thinking, knowledge in childhood obesity prevention, and evidence-based actions across community networks to drive sustainable PSE change [3, 12]. In practice, CCI brings together new or existing multisectoral committees to participate in Group Model Building sessions that foster a shared understanding of the complex systems contributing to childhood obesity in their community [13]. These sessions help map local factors influencing child health and identify opportunities for PSE change. Committees receive seed funding to implement selected PSE actions and ongoing technical assistance from the research team in systems thinking, meeting facilitation, and PSE implementation, evaluation, and strategy refinement, with the goal of building community capacity to enact sustainable, equity-oriented change [14].

To date, findings from the CCI reveal four key outcomes across diverse community settings. First, participants consistently deepened their systems thinking, shifting from individual-level views to recognizing the structural and systemic drivers of child health, such as racism, socioeconomic inequities, and community disinvestment [3, 12]. Second, these systems insights translated into stronger equity-focused action as stakeholders increasingly identified leverage points to reduce deterrents to health and expand community capacity [14]. Third, the initiative fostered inclusive and strategic collaboration, with coalition members building trust, shared leadership, and interorganizational alignment through Group Model Building and facilitation processes [8, 10]. Finally, changes in coalition structure and network connectivity supported the diffusion of knowledge and engagement, though discrepancies in perceived influence in their social network and participation in the coalition remained for less-experienced or peripheral members [9, 10].

This evaluation reports the PSE impacts of PSE-related actions in three diverse U.S. communities participating in CCI. “PSE-related actions” refer to the discrete policy, systems, or environmental strategies initiated or advanced through the CCI, as reported by participants in the surveys. The specific actions implemented by committees have been described elsewhere [14]. Briefly, these include actions such as the creation of communication materials about food assistance programs and hosting health events. “PSE impacts” refer to perceived changes, ripple effects, or secondary conditions attributed to those actions, such as shifts in partnerships, funding, or organizational practices. We use the term ‘outcomes’ only when referring generically to the results of PSE change, not as a substitute for ‘impacts.’

This analysis integrates three complementary frameworks and tools. The Getting to Equity (GTE) framework provides a structured way to categorize and interpret strategies that advance equity, especially as it relates to obesity prevention [5, 15]. The Consolidated Framework for Implementation Research (CFIR) identifies contextual factors that influence implementation across multiple levels [16]. Whereas Group Model Building and causal loop diagramming (CLD) supported committees’ planning and systems understanding during the CCI, REM was used in this evaluation phase to examine how PSE impacts unfolded and interacted across contexts. Ripple Effects Mapping (REM), a systems science approach, visualizes the indirect and reinforcing effects of interventions over time by linking participant narratives to causal pathways [17]. Integrating these tools allowed us to examine both the content and conditions of equity-focused PSE change: the GTE framework classified the nature and equity focus of PSE-related impacts, CFIR identified multilevel contextual factors shaping their implementation, and REM captured the indirect and reinforcing dynamics through which those impacts diffused and sustained PSE change across community systems..

The intent of these analyses is to generate findings relevant to diverse actors and stakeholders currently or potentially engaged in equity-focused whole-of-community childhood obesity initiatives through PSE change. We also demonstrate the value of using relevant frameworks to analyze and interpret findings in a complex system.

## Methods

### Evaluation Design

This evaluation used a mixed-methods design to examine PSE changes that emerged from the SDCD theory-informed CCI in three communities. Guided by the GTE framework, CFIR, and REM, the analysis focused on: (1) identifying the types and implementation stages of committee actions; (2) assessing reinforcing synergies among PSE impacts resulting from committee actions; (3) visualizing ripple effects of such impacts within and between communities; and (4) examining contextual factors that influenced PSE change. The initiative was approved by the [redacted] Institutional Review Board. This multi-framework approach reflects the complexity of community-driven systems change, where identifying what types of PSE impacts occur (GTE), understanding why they occur (CFIR), and visualizing how they reinforce one another (REM) are interdependent.

The GTE framework provided the central lens for classifying and interpreting PSE impacts [5, 15]. GTE conceptualizes equity-oriented change across four quadrants: (1) increasing healthy options, (2) reducing deterrents to healthy behaviors, (3) improving social and economic resources, and (4) building on community capacity and opportunity. These quadrants are interrelated and can reinforce one another through synergistic influence. Applying GTE enabled classification of PSE-related impacts both by their target domain and interactions across quadrants to advance equity. The framework’s emphasis on synergy aligns closely with the CCI’s systems-based design and provided a foundation for subsequent REM and contextual analysis. The GTE has been applied to interventions such as online food shopping for SNAP participants [18, 19], the food banking system [20] and universal school meals [21].

The CFIR guided identification of contextual factors shaping PSE change at the individual (leadership, motivation), inner-setting (committee processes, facilitation quality), and outer-setting (policy, partnerships, and socioeconomic environment) levels.

REM was used to capture the dynamic interactions among PSE impacts and their downstream ripple effects. Information on ripple effects was derived inductively from interview narratives describing secondary or unintended consequences of initial impacts. These instances were extracted, coded, and iteratively connected to visualize reinforcing and counteracting dynamics across GTE quadrants.

### Data Sources and Sampling Frame

Data were collected from two primary sources: surveys and qualitative interviews with CCI participants. The initiative included 39 committee members, of whom 35 participated in the present evaluation: Community 1 (*n* = 11), Community 2 (*n* = 12), and Community 3 (*n* = 12). Participants completed surveys and participated in interviews one year after the start of CCI in 2023. The committees across the three communities were selected through a competitive request for partnership process. Applicants were existing coalitions and were asked to describe their existing community partnerships, leadership structure, and interest in advancing PSE change related to child health and obesity prevention. They also provided information on community demographics, local priorities, and capacity to engage in systems-based work. The selection process prioritized diversity in geography, population demographics, and focus areas in obesity prevention, while ensuring that all selected coalitions began the CCI at approximately the same time to allow for comparability across sites.

Participants represented a range of sectors central to child health and community wellbeing, including public health departments, healthcare organizations, early childhood education, school systems, local government, nonprofit organizations, and community-based food access and social service groups. Each committee aimed to ensure representation from multiple sectors influencing the social determinants of health and childhood obesity. Participants contributed perspectives grounded in their professional expertise, organizational roles, and lived experience within the community. Committees were constituted through purposive recruitment by coalition leads to capture a balance of decision-makers, program implementers, and resident representatives.

### Survey Measures

Survey development drew on prior evaluation tools from the Healthy Communities [22], an early childhood PSE systematic review [23], the National Comprehensive Cancer Control Program [24], and the Oklahoma Cooperative Extension survey [25]. These instruments guided the selection, wording, and adaptation of items assessing local actions for PSE change related to childhood obesity prevention. The final survey included four domains: (1) identification and description of PSE-related actions (up to three per participant) that the committee prioritized for implementation during the CCI, (2) stage of implementation for each action (planning, initiated/in progress, completed, sustained), (3) committee involvement (supported, facilitated, or led by the CCI committee), and (4) perceived facilitators and barriers to implementation. Example questions included: “List up to three policy, systems, or environmental actions your organization implemented during the Catalyzing Communities Initiative,” and “At what stage is each action currently?” The survey also included the question, “What about participating in the Catalyzing Communities committee made implementing PSE change easier?” to capture participants’ reflections on facilitators of implementation. Responses were analyzed descriptively and integrated with interview data during triangulation to deepen understanding of actions’ implementation, contextual influences, and perceived impacts of such actions.

### Qualitative Interviews

In-depth, semi-structured interviews were conducted with participants (*n* = 29) who had completed the survey approximately one year after the intervention began. These interviews were designed to capture longitudinal insights into how PSE impacts developed as a result of committee actions, and evolved over time, and what factors influenced PSE change. Participants were asked about their personal views regarding the causes of unhealthy child weights and the relationship between food security, child health equity, and healthy weights in their communities. They identified priority actions for promoting healthy child weights and discussed potential barriers to implementing those actions. Additional questions explored actions taken as a result of committee participation, impacts on their organizations, and perceived effects on the broader community. The interviews served as the primary data source for the analysis of types of PSE impacts, their ripple effects and synergies, as well as contextual influences.

The six participants who did not complete interviews were evenly distributed across each of the three communities and primarily represented organizational partners rather than resident leaders. Their nonparticipation was attributed to job turnover and scheduling challenges during the data collection period. While their perspectives may have provided additional insight into organizational facilitators and barriers, triangulation of survey and interview data helped ensure that themes reflected the full range of perspectives captured through the committee network.

### Data Analysis

We used convergence coding to triangulate survey and interview data. Survey data were analyzed descriptively, and the implementation stage was documented. Survey responses were compared with interview content to validate and expand on reported PSE-related changes.

Interview transcripts were coded thematically using a structured codebook developed through an iterative process combining inductive and deductive approaches (Supplementary Table S1). Coding was guided by the GTE framework, the SDCD framework, and CFIR. Two trained coders independently coded transcripts using Dedoose software. Intercoder reliability was established through discussions and by resolving discrepancies; Cohen’s Kappa values indicating at least substantial agreement (κ = 0.61) served as a guideline for identifying areas needing further review or discussion. Code frequencies and co-occurrences informed the identification of PSE impacts, what GTE quadrant they mapped onto, their status, synergies, and contextual factors.

Using interview data, we applied REM to identify downstream PSE impacts and visualize how initial impacts generated secondary or reinforcing ripple effects [17, 21]. Coders extracted statements describing follow-on impacts and linked them conceptually through causal chains reflecting how one change influenced another. The analysis focused on identifying reinforcing and balancing feedback loops that illustrate the mechanisms through which community capacity, social connections, and structural conditions interacted to sustain or constrain PSE change. We defined synergy as the interaction of PSE impacts across GTE quadrants in ways that reinforced, amplified, or extended the effects of individual PSE-related actions. Synergy was identified by examining clusters of impacts that participants described as mutually enabling or cascading, and by mapping how these clusters spread across domains using REM in Kumu software, a web-based system visualization platform. We then grouped related clusters into themes that reflected recurring cross-quadrant patterns. The total counts reported in results reflect the number of times each impact was mentioned across participant interviews, not the number of unique impacts.

## Results

### Characteristics of Committee Members and Communities

Three community-based committees participated in this evaluation, one from each of the three communities. To protect participant confidentiality, individual demographic data are not reported; however, Table 1 summarizes key characteristics of each community and its corresponding committee.

**Table 1:** Summary of community and coalition-committee characteristics

Community	1	2	3
**Community characteristics**^**a**^			
Population estimate	205,319	64,670	19,992
Land area (mi^2^)	37.36	20.33	1.94
Median household income (USD)	$56,746	$36,184	$23,067
Foreign born (%)	21.9	41.8	2.1
**Community Race and ethnicity (%)**			
Hispanic or Latino (all races)	23.9	85.2	4.8
NH White	53.6	11.0	5.9
NH Black or African American	12.7	2.7	84.9
NH American Indian and Alaska Native	0.5	0.9	0.1
NH Asian	6.8	0.7	0.9
NH Native Hawaiian and Other Pacific Islander	0.1	0.9	0.1
NH some other race	0.1	0.8	0.1
NH two or more races	9.8	6.9	3.3
**Committee characteristics**			
Committee size (n)	11	14^d^	14^d^
Bachelor’s degree and above (%)	66.7	71.4	50.0
Female (%)	81.8	71.4	100.0
Target child age	0–19	3–19	0–19
Committee Focus Area(s)^b^	Decrease food insecurity through mitigating impact of cliff effect and increasing equitable funding distribution	Community-wide, multi-sector movement focused on access to and knowledge of healthy food, supporting active lifestyles, and promoting community belonging	Increase access to and consumption of healthy foods through produce prescription programs and increase community connection through communal meals

^a^American Community Survey, 2019

^b^Focus areas were determined through group model building activities and the prioritization of actions to be carried out by the committee all occurred in year 2023

^c^American Community Survey, 2020

^d^These two committees started with 14 members, 12 of whom remained throughout the Catalyzing Communities initiative and participated in the present study; percentages of committee characteristics are based on baseline participation. Thus, the total sample size for this study is 35

### Types and Statuses of PSE Changes by GTE Quadrant

**Fig. 1:**
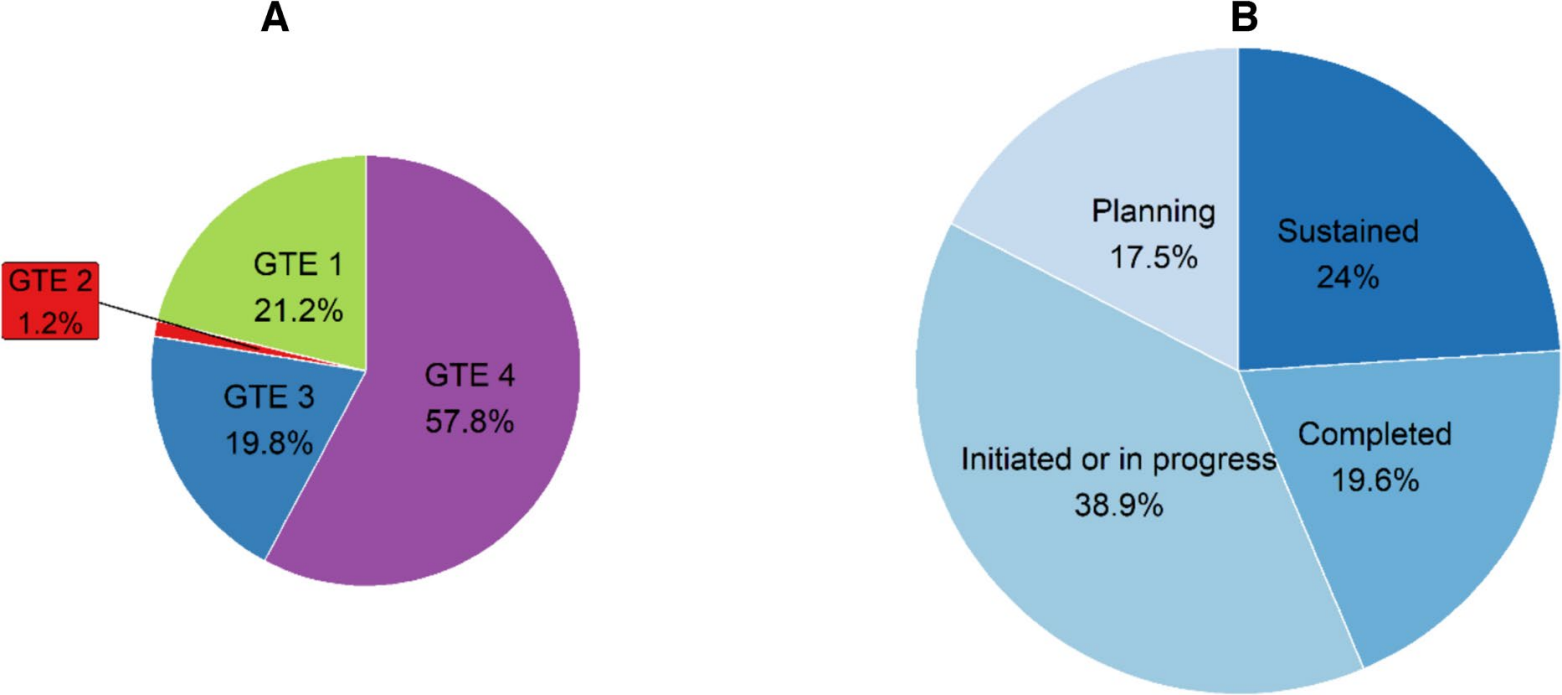
Frequency of PSE impacts by GTE quadrant (**A**) and their status (**B**). See Supplementary Table S2 for examples and full quote data. *Note*. Panel A categorizes PSE impacts by GTE quadrant: (1) increasing healthy options, (2) reducing deterrents, (3) improving social and economic resources, and (4) building on community capacity. Panel B shows the status of such impacts: planning (idea development), initiated or in progress (actively underway), completed (fully achieved), and sustained (maintained over time)

Across the three communities, 29 committee members reported a total of 339 PSE impacts. The frequency of each impact reflects how often it was mentioned across interviews. The majority of reported impacts (*n* = 196; 58%) aligned with GTE Quadrant 4 (Build on Community Capacity), followed by Quadrant 1 (Increase Healthy Options; *n* = 72; 21%), Quadrant 3 (Improve Socioeconomic Resources; *n* = 67; 20%), and Quadrant 2 (Reduce Deterrents; *n* = 4; 1%) (Fig. 1A). The status of these impacts was reported for 275 of the 339 impacts. Of these, most were in the Initiated/In Progress phase (*n* = 107; 39%), followed by Sustained (*n* = 66; 24%), Completed (*n* = 54; 20%), and Planning (*n* = 48; 17%) (Fig. 1B). The status could not be determined for 64 of the 339 reported impacts because participants described the impacts conceptually rather than operationally, making their status unclear (e.g., “community collaboration” or “local food access”). These entries were retained in the overall impact total but excluded from status analysis.

Although participants could list up to three PSE-related actions in the survey, additional actions emerged from qualitative interviews. Perceived impacts of all actions were coded and counted, yielding a total of 339 reported impacts. This number reflects the number of times they were mentioned rather than unique impacts, as some were cited by multiple participants or repeated by the same participant. All reported impacts were reviewed and classified using the GTE framework, and each was deemed to advance at least one equity-related goal, such as improving access, opportunity, or community capacity.

### Synergy of PSE Changes Across GTE Quadrants

Ten themes emerged that reflect how PSE impacts were synergistic across GTE quadrants (See Supplementary Table S3 for themes and quotes). Most common were synergies between GTE Quadrants 1 (Healthy Options) and 4 (Community Capacity) (*n* = 5), where systems thinking and increased health promotion reinforced PSE change. For example, the acquired skill to create (GTE 4) was reported to strengthen health initiatives and advocacy for healthier food environments (GTE 1). Synergies between Quadrants 1 and 3 (Social and Economic Resources) (*n* = 2) involved the ability to leverage social and financial capital (GTE 3) to strengthen community-led health initiatives (GTE 1). Others, such as between Quadrants 1 and 2 (Reduce deterrents) (*n* = 1), reflected reframing of physical activity (GTE 1) to reduce weight stigma (GTE 2). Across Quadrants 2 and 4 (*n* = 1), enhanced systems thinking capacity (GTE 4) was reported to help identify and contextualize structural barriers (GTE 2). Lastly, across Quadrants 3 and 4 (*n* = 1), the increased social capital and, in particular, the resources shared through social connections (GTE 3) further strengthened community capacity to work collaboratively (GTE 4).

### Ripple and Reinforcing Effects of PSE Change Across Communities

**Fig. 2:**
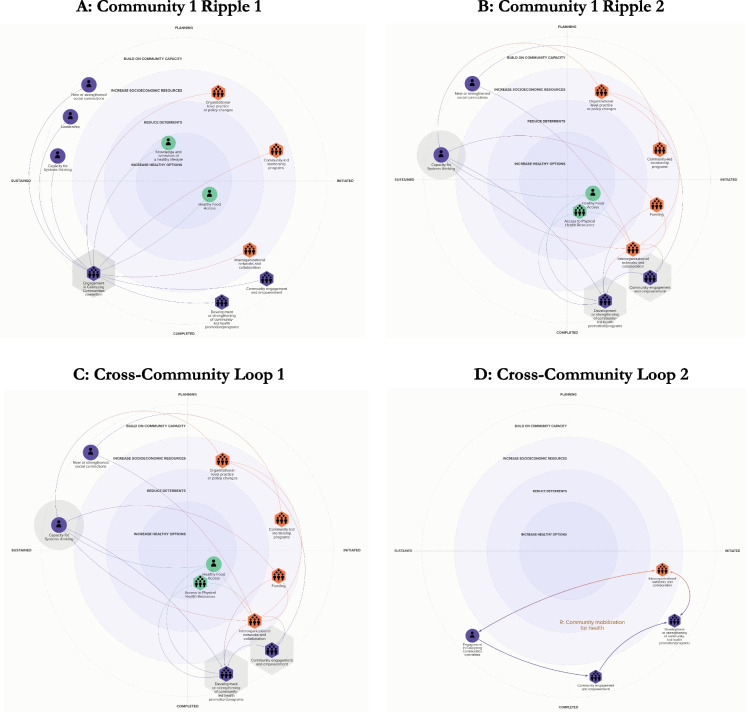
Illustrating Ripple Effects of Policy, System, Environmental Change Actions in Community 1 (**A**, **B**). Feedback Loops Across Communities (**C**, **D**). (See Supplementary Figure S1 for ripple effects for communities 2 and 3). *Note*. Panels **A** and **B** are ripple effects of PSE impacts in Community 1 after participating in the CCI committee. For the ripple effects in Communities 2 and 3, please see the supplemental material. Panels **C** and **D** are feedback loops that emerged across the three communities. All effects are positive

As shown in Fig. 2(A and B), participation in the CCI served as a foundation for a wide range of ripple effects across all three communities. Early impacts included strengthened systems thinking capacity, deeper social connections, enhanced leadership (GTE 4), and initial organizational policy and program shifts (GTE 3: Increase Socioeconomic Resources). These, in turn, contributed to improved access to healthy food (GTE 1: Increase Healthy Options), sustained health promotion efforts, and the development of culturally responsive, community-led initiatives (GTE 4).

Across communities, three consistent themes emerged. First, participation in the CCI built systems thinking skills (GTE 4) that directly informed strategic organizational changes, often resulting in more inclusive food access initiatives and increased alignment with health-related efforts (GTE 1, GTE 3). Second, social connections formed through the CCI fostered interorganizational collaboration and broader diffusion of practices (GTE 4, GTE 3). Third, community-led programs, often designed with resident input, became central mechanisms for sustaining and reinforcing change (GTE 4).

Two reinforcing feedback loops, shown in Fig. 2 (C and D), illustrate how these dynamics unfolded. The first loop, systems-informed community action, begins with committee participation strengthening systems thinking (GTE 4), leading to organizational change (GTE 3) and health-supportive environments (GTE 1), which in turn drive further engagement and action (GTE 4). The second loop, community mobilization for health, involves deeper resident engagement (GTE 4), the co-creation of culturally relevant initiatives (GTE 1), and the attraction of new partners and resources (GTE 3), reinforcing both programs and committee functioning (GTE 4).

Though patterns of synergy were consistent, each community exhibited unique ripple effects. In Community 1 (C1), recurring community meals increased food access (GTE 1), served as hubs for civic dialogue and grant development (GTE 3), and strengthened community engagement (GTE 4). Community 2 (C2) emphasized information diffusion and outreach (GTE 4, GTE 3), with health promotion efforts catalyzing collaboration and leadership development emerging from shifting norms around health behaviors (GTE 4). In Community 3 (C3), committee engagement generated additional actions to build on community capacity (GTE 4), socioeconomic resources in the form of interorganizational networks, funding, and policy changes (GTE 3), and access to healthy options (GTE 1).

### Contextual Factors Reinforcing or Counteracting PSE Changes

Contextual factors altered PSE change broadly, encompassing factors that influenced the implementation of committee actions or the resulting PSE impacts. Interviews revealed a range of contextual factors influencing PSE change, aligning with the CFIR across three levels: individual, inner setting (committee), and outer setting (organizations and communities). These factors either reinforced or impeded progress toward health equity, as seen in Table 2.

**Table 2:** Contextual factors influencing PSE change, by CFIR level and synergy type. See Supplementary Table S4 for additional quotes

CFIR level	Contextual factor	Synergy type	Illustrative quote
Individual	Prior committee/taskforce participation	Reinforcing	*As a representative of the school district, I serve as a school board member, I have shared along the way with the other trustees what the committee has been doing*
Individual	Leadership skills and initiative	Reinforcing	*It's like you know how you have a little fire, right? You light this little fire like a candle. It's a little fire. It feels good. It's warm. It makes sense. That's where I was as far as nutrition and my discussions with my clients at first. As a result of being around other people on the team and talking to other people on the stakeholder committee, I'm like, "No, this needs to be a forest fire. This is a bigger conversation that needs to happen*
Inner Setting	Committee facilitation	Reinforcing	*[Research team member’s name redacted] is just amazing. She kept it all together and she keeps you focused and she draws from you and she makes you feel important in whatever little tidbit you have. She could brighten it up for everybody. It was really great*
Inner Setting	Cross-organizational resource exchange	Reinforcing	*We're all reminding each other of different sectors that are coalescing together and that, again, reinforcing the fact that they all play together*
Outer Setting	Tangible/financial supports	Reinforcing	*The committee and the action plan that we decided upon, that brought change and it's growing from there. Having someone else [i.e., Research team] come in and help us with the meals and seeing that it's important so they're going to help and pick up with funding*
Outer Setting	Pre-existing programs and partnerships	Reinforcing	*There has always been that push for health and wellness in the organization. I think with, with this Committee, it's like move it now, like there is urgency in getting things accomplished and relationships and events going*
Outer Setting	Local policy context	Reinforcing	*I think the school district and our government, our leadership, need to get together and work together to [promote physical activity]*
Outer Setting	Community unsafety	Counteracting	*The violence has plagued our community so bad to where people be scared to even come outside to go to the grocery store*
Outer Setting	Stigma	Counteracting	*There's still a lot of stigma to food banks. I know better because I did food distribution, so I'm like, "You got to know where to get it." Not every family sees it the same way. There's a lot of pride involved*
Outer Setting	Low affordability of food and physical activity	Counteracting	*We have [health centers that promote physical activity], but the parents can't afford to go*
Outer Setting	Commercial food interests	Counteracting	*In this area, all you see is fast food locations. […] Do you see a grocery store? No, we don't. We don't have that. That definitely contributes to child obesity*

At the individual level, prior committee experience and strong leadership skills were perceived by members to help mobilize networks and initiate new programs. Within committees, effective facilitation and cross-sector collaboration fostered trust, alignment, and momentum. Outer-setting factors such as stipends, meals, pre-existing partnerships, and supportive local policies further sustained action. However, counteracting forces (reported exclusively in the outer setting) included community violence, stigma around food assistance and weight, limited affordability of healthy options, and the dominance of commercial food outlets.

Survey responses echoed these findings. Among 35 participants, 62% cited increased awareness of local programs and resources as the most helpful factor in implementing PSE changes. Others highlighted the value of facilitated group meetings (16%), developing CLDs (12%), and working on concrete actions (10%). Table 2 summarizes these contextual influences by CFIR level and synergy type. Supplementary Table S4 provides additional participant quotes.

## Discussion

Whole-of-community approaches that aim to advance child health equity through PSE change face persistent challenges in settings shaped by structural barriers to health [5, 26]. This evaluation examined how the CCI, informed by Stakeholder-Driven Community Diffusion [8], supported equity-focused PSE change across three communities [22]. Broadly, the main findings are that: (1) most perceived impacts mapped to the GTE quadrants that increase healthy options and build on community capacity; (2) PSE impacts interacted across quadrants in ways that created reinforcing patterns of equity-oriented change; and (3) contextual factors at individual, committee, and community levels either amplified or constrained these dynamics.

These findings extend prior work on community coalitions and systems-informed childhood obesity prevention [3, 8, 10, 12, 14, 27]. Earlier evaluations described how CCI deepened systems thinking, strengthened collaboration, and advanced PSE-related actions [3, 12, 14, 27]. The present study builds on that work by tracing how those actions translated into perceived PSE impacts across equity domains, how impacts interacted to create synergy, and how context shaped those patterns. The combined use of GTE, CFIR, and REM also provides a way to examine what changed, why it changed, and how changes appeared to reinforce one another. This tri-framework approach contributes a practical strategy for unpacking mechanisms in community-driven, systems-oriented interventions where traditional linear evaluation models may be insufficient.

### Patterns of PSE Change Across GTE Quadrants

Most PSE impacts fell within GTE Quadrant 4 (building on community capacity) and Quadrant 1 (increasing healthy options). Capacity-building impacts included stronger cross-sector relationships, improved systems thinking, and enhanced committee functioning. Increases in healthy options included expanded access to culturally relevant food, health-promoting programming, and supportive physical activity environments. This aligns with prior CCI studies and with other whole-of-community obesity prevention efforts, which often report early gains in leadership, collaboration, and health-promoting environments [3, 6, 8, 14, 21].

The finding that fewer impacts mapped directly to Quadrant 2 (reducing deterrents to healthy behaviors) and Quadrant 3 (improving socioeconomic resources) likely reflects both the limited time frame of the evaluation and the nature of the actions that coalitions were able to advance within one year. Resource redistribution and deterrent reduction often require longer time horizons, policy change, and shifts in economic or social conditions. In contrast, changes in programming, partnership, and community engagement can emerge more rapidly. However, ripple effects suggested that Quadrant 1 and 4 strategies may create conditions that support later movement in Quadrants 2 and 3. For instance, community meals and outreach activities (Q1 and Q4) were described as creating spaces for civic dialogue, grant development, and policy discussions that could influence affordability or stigma over time (Q2 and Q3).

Classification within the GTE quadrants required judgment in cases where impacts could plausibly fit multiple domains. For example, some initiatives both increased access to healthy options and reduced social or logistical barriers to their use. In those cases, coders relied on participants’ descriptions of the primary mechanism of change to assign impacts to a single quadrant. This approach is consistent with guidance for applying GTE in complex interventions [5, 21] while acknowledging that equity strategies often span multiple domains. The need for such classification decisions highlights an area where future work could further refine how GTE is operationalized in dynamic community settings.

Several impacts described organizational shifts, such as changes in staffing, decision-making processes, or internal alignment around health equity. These are not PSE changes in a strict sense. They were included when participants linked them to community-level or structural consequences, such as increased capacity to pursue policy change, expand services, or coordinate across systems. In that context, organizational adaptations functioned as precursors that helped coalitions move from isolated programs toward more systemic action. This distinction supports the growing emphasis on mechanisms that connect internal organizational change to external equity outcomes [4, 28, 29].

### Synergy and Feedback Dynamics

Synergy, defined in this study as reinforcing interactions among PSE impacts across GTE quadrants, emerged as a central mechanism through which CCI-related actions appeared to support equity-oriented change. This is illustrated by the findings from ripple mapping showing how early gains in systems thinking, leadership, and collaboration (Q4) linked to shifts in organizational practices and resource flows (Q3), which in turn supported increased access to healthy options (Q1) and sustained community engagement (Q4). These interactions generated reinforcing feedback loops, such as systems-informed community action and community mobilization for health, that operated across all three communities.

CLDs and Group Model Building helped committees recognize leverage points and align actions with local drivers of child health. This often led to Q1 community-led initiatives such as recurring community meals, culturally tailored outreach, or resident-led walking groups. These initiatives then deepened relationships, trust, and shared purpose, consistent with Q4 concepts of social capital and collective efficacy [30–32]. In several cases, new partners and funding opportunities emerged from this momentum, indicating that capacity-building and access-enhancing strategies can help attract socioeconomic resources (Q3) that further reinforce equity-oriented change.

Synergies that involved Quadrant 2 and Quadrant 3 were less frequent but conceptually important. Participants described instances where systems thinking helped contextualize structural barriers (deterrents), such as stigma or community violence, and where expanding networks and visibility created opportunities to address affordability or resource gaps. These impacts were early and often indirect, but they illustrate how PSE change in one domain may open pathways into deeper structural work. Future CCI iterations could build on these insights by designing explicit cross-quadrant strategies that pair capacity-building and access-focused efforts with actions that reduce deterrents and redistribute resources.

### Contextual Conditions Shaping PSE Change

Inner-setting dynamics appeared less prominent in the data than outer-setting factors. One likely reason is that the CCI committees functioned as multi-organizational bodies rather than single organizations. Participants often anchored their narratives in their home institutions or the broader community environment rather than in the committee as a discrete organizational setting. Additionally, the interview guide focused more on perceived impacts and community context than on internal committee processes, which may have limited the detail available about inner-setting constructs.

Outer-setting conditions exerted a strong influence on whether and how PSE changes unfolded. Supportive factors included existing partnerships, availability of seed funding, alignment with local policy priorities, and concrete supports such as stipends and meals that reduced participation barriers. At the same time, participants highlighted constraints such as community violence, stigma related to food assistance and weight, limited affordability of healthy options, and the density of commercial food outlets. These conditions shaped both the feasibility and perceived reach of PSE-related actions. The concentration of barriers in the outer setting reinforces calls to examine how structural determinants of health act as both targets and constraints for implementation strategies [10, 24, 33].

### Methodological Contributions and Conceptual Boundaries

Integrating GTE, CFIR, and REM provided a practical framework for examining equity-oriented PSE change in community coalitions. GTE offered a shared language to classify impacts and assess whether they aligned with equity-oriented goals [5, 15]. CFIR supported the systematic identification of contextual influences at multiple levels [16]. REM complemented both by revealing how participants perceived impacts to accumulate, diffuse, and reinforce one another over time [17]. Together, these tools helped move beyond cataloging isolated actions and toward understanding patterns of change that are consistent with systems perspectives on community interventions.

At the same time, the scope of inference from this evaluation remains conceptual rather than causal. The tri-framework approach illuminates plausible pathways and perceived mechanisms through which CCI participation may influence PSE impacts and equity-related outcomes. It does not test the SDCD theory or quantify system behavior. The findings should therefore be interpreted as an early-stage evaluation of perceived impacts and system dynamics that can inform theory refinement and future mixed-methods or modeling work, rather than as evidence that specific feedback loops will reliably emerge in other contexts.

### Public Health Implications

The findings suggest several implications for practitioners, funders, and policymakers seeking to support equity-focused PSE change through community coalitions. First, investing in systems thinking capacity and structured facilitation appears to help coalitions translate lived experience and local data into coordinated PSE actions. Tools such as CLDs and REM can support coalitions in identifying leverage points, tracking ripple effects, and adjusting strategies in response to emerging patterns.

Second, coalitions may achieve early gains by focusing on actions that both increase healthy options and build community capacity. Examples from this evaluation include community meals that serve as hubs for connection, culturally responsive outreach, and resident-led programs. These actions appeared to generate reinforcing loops that deepened engagement, attracted partners, and created momentum for additional change. Designing interventions that intentionally cultivate these loops may strengthen the durability and reach of equity-focused efforts.

Third, equity goals require explicit attention to Quadrants 2 and 3, including deterrent reduction and resource improvement. The relatively limited number of impacts in these domains underscores the structural complexity of affordability, stigma, and socioeconomic inequities. Whole-of-community initiatives can help prepare the ground for such work by building relationships, legitimacy, and local evidence. However, progress in these quadrants likely depends on aligning coalition efforts with policy advocacy, institutional reforms, and financing strategies that extend beyond the scope of community programming alone.

Fourth, this evaluation illustrates the need for detailed information about coalition activities and participant experiences. Generating insights about how PSE actions unfold requires time, depth, and multiple perspectives; these elements strengthen the credibility of findings even when recall or reporting biases are possible. Building structured checks and balances into routine data collection can help coalitions maintain this level of rigor while keeping the process feasible.

Fifth, evaluation approaches that integrate equity frameworks with implementation and systems science can help practitioners and decision makers understand not only whether PSE actions are implemented, but how and under what conditions they contribute to equity-oriented change. Routine use of tools such as GTE, CFIR, and REM in community practice settings could support adaptive learning, help coalitions identify gaps across equity domains, and inform resource allocation toward strategies that have reinforcing potential. Relatedly, although CCI centered on childhood obesity, committee members pursued varied priorities, and the same systems-oriented, equity-focused approach could support coordinated PSE action in other public health areas. This suggests that the combined use of GTE, CFIR, and REM is transferable when adapted to local context and goals.

### Limitations and Directions for Future Research

Several limitations should guide interpretation of these findings. Data were drawn from three communities that had the capacity and interest to participate in CCI, which may limit transferability to other settings. The evaluation relied on self-reported surveys and interviews, which are subject to recall and social desirability bias. Ripple effects and feedback loops were identified through qualitative analysis rather than direct observation or formal modeling and thus reflect participants’ perceptions of system change at an early stage.

Coding decisions, including assignment of impacts to single GTE quadrants and distinctions between organizational and community-level change, required judgment and may not capture all nuances of equity-relevant action. Inner-setting constructs within CFIR were less fully described than individual and outer-setting factors, partly due to the design of the interview guide and the multi-organizational nature of the committees. The evaluation period was relatively short, which constrained the ability to observe longer-term structural change in Quadrants 2 and 3 or to follow the full trajectory of identified feedback loops.

Future research could address these limitations by extending follow-up periods, incorporating routine or administrative data on PSE outcomes, and using mixed-methods designs that integrate REM with social network analysis, system dynamics modeling, or other quantitative systems approaches. Comparative studies across a larger number of communities could examine how different configurations of contextual factors and coalition structures influence the emergence of synergy across GTE quadrants. Methodological work is also needed to refine how GTE and CFIR can be jointly applied to equity-focused, community-driven interventions and to develop guidance for distinguishing organizational precursors from PSE change while still capturing their role in equity pathways.

Within the CCI context, next steps could include co-developing strategies with community partners that more directly target deterrent reduction and resource redistribution, documenting how coalitions navigate structural constraints, and examining how shifts in power, voice, and representation within coalitions relate to perceived and observed PSE impacts. Such efforts would help deepen understanding of how community-led, systems-informed interventions can contribute to structural change that advances child health equity.

## Supplementary Information

Below is the link to the electronic supplementary material.

Supplementary file1 (DOCX 3149 kb)

Supplementary file2 (DOCX 28 kb)

Supplementary file3 (DOCX 17 kb)

Supplementary file4 (DOCX 30 kb)

Supplementary file5 (DOCX 21 kb)

## Data Availability

All data generated or analysed during this study are included in this published article, with the exception of the qualitative interview dataset. The qualitative data are openly available through the Open Science Framework at https://osf.io/fhk94.
