# Supplementary material for: Identifying Synergistic Mechanisms of Community-Led Policy, Systems, and Environmental Change for Childhood Obesity Prevention in the Multi-Site Catalyzing Communities Initiative
Source: J Urban Health. 2026 Jan 7;103(1):77–91. doi: 10.1007/s11524-025-01046-y (PMC13136475; doi:10.1007/s11524-025-01046-y)

**Supplementary Figure S1**. Illustrating Ripple Effects of Policy, System, Environmental Change in Communities 2 (Panels A, B) and 3 (Panels C, D)

**Panel A: Community 2 Ripple 1** **Panel B: Community 2 Ripple 2**


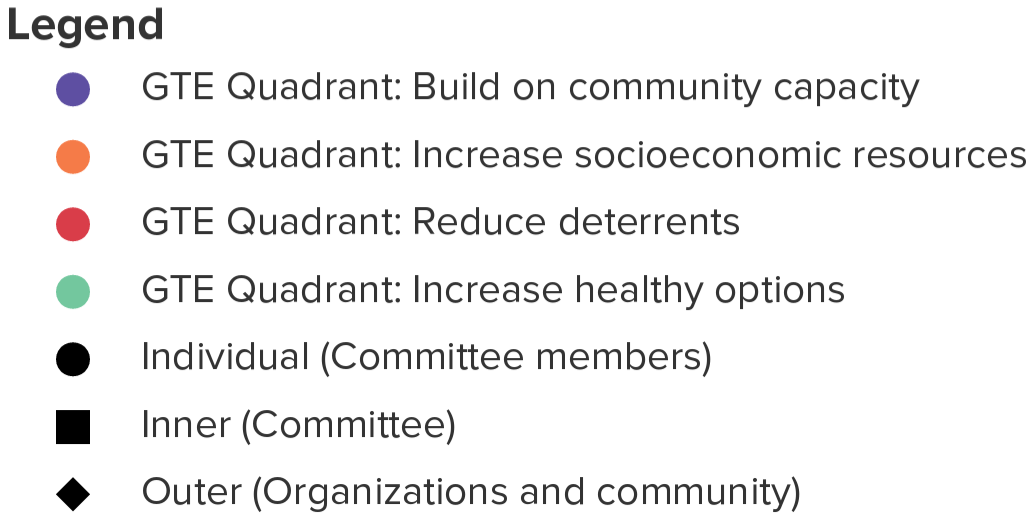

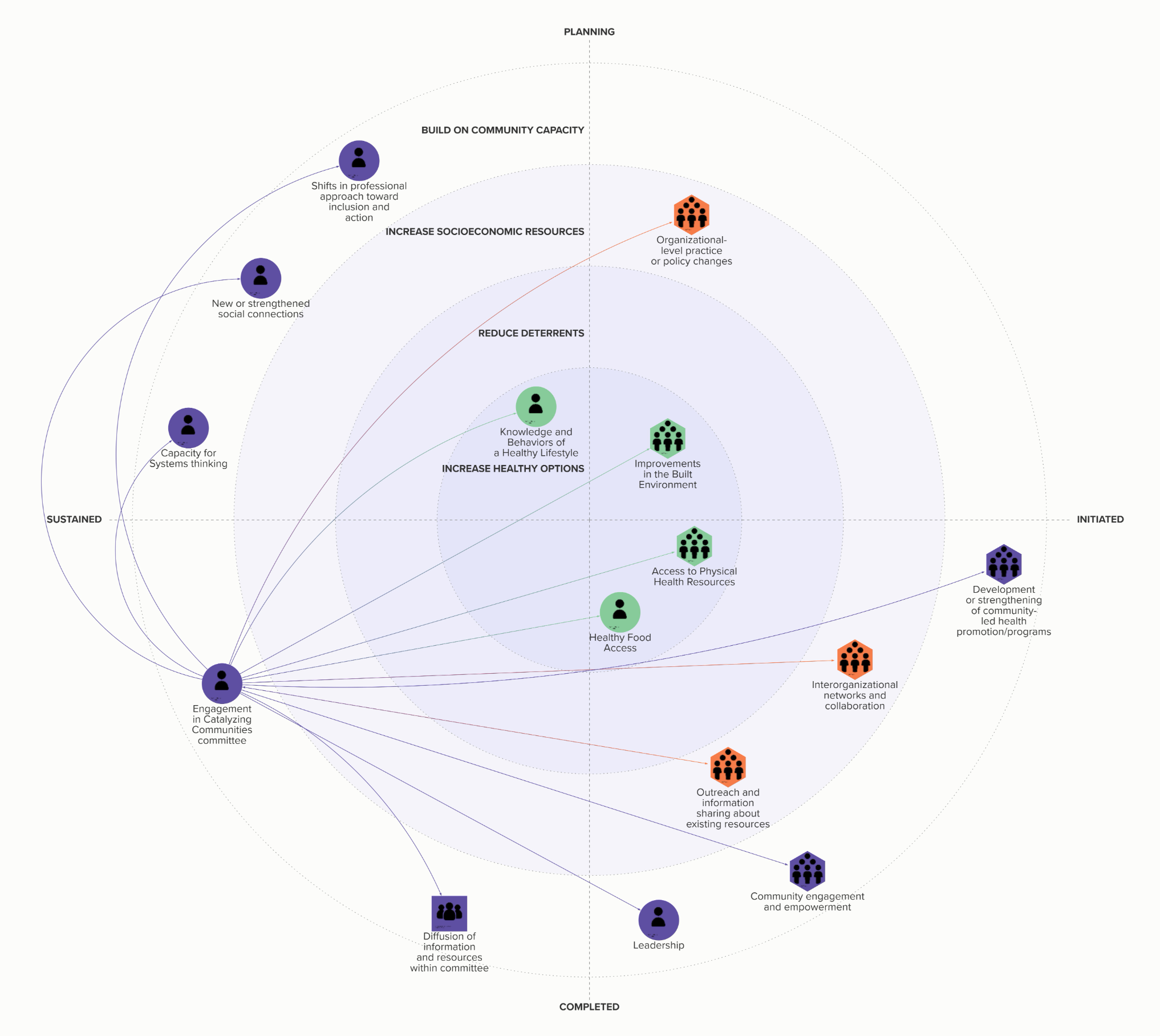

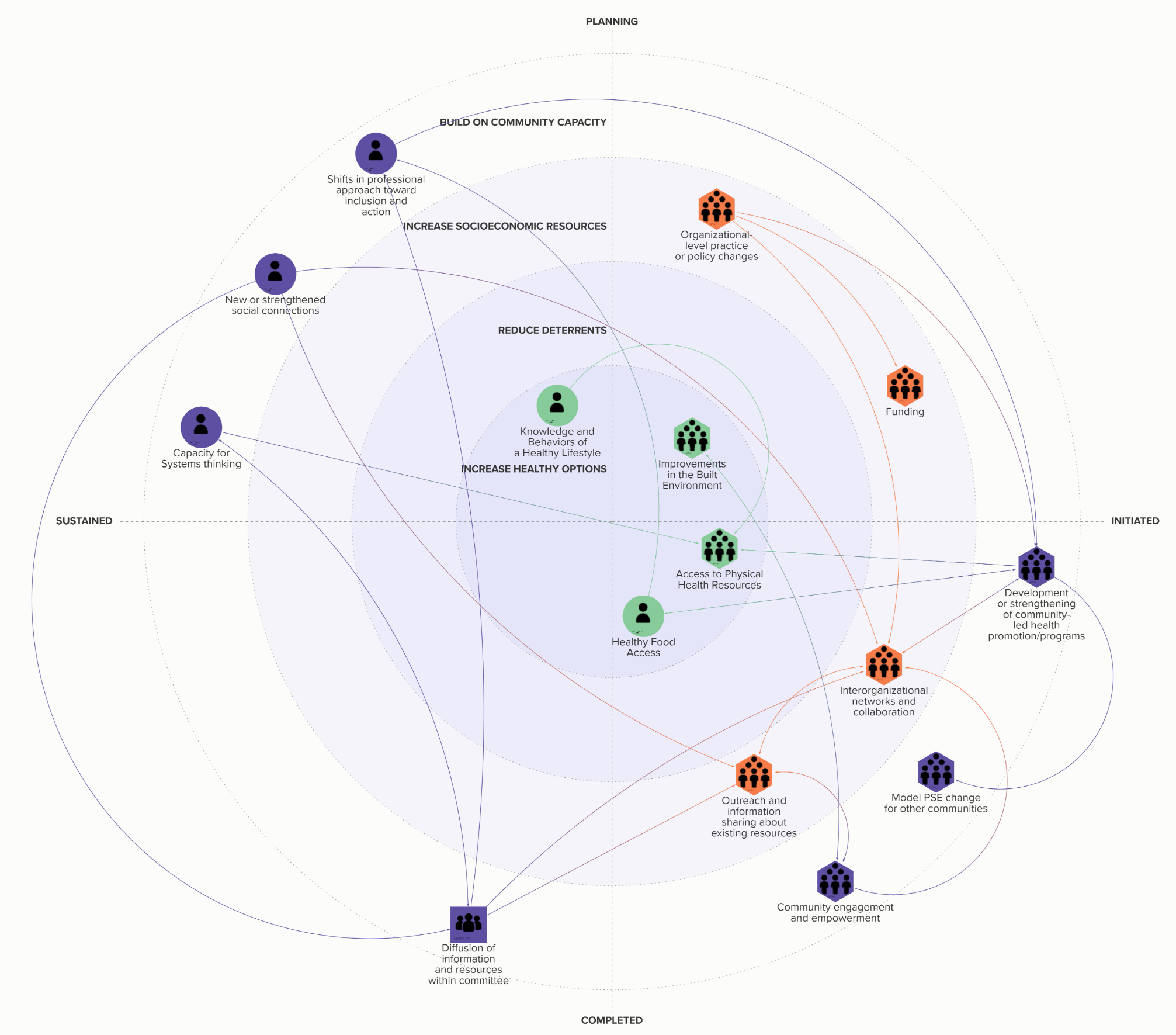


**Panel C: Community 3 Ripple 1** **Panel D: Community 3 Ripple 2**


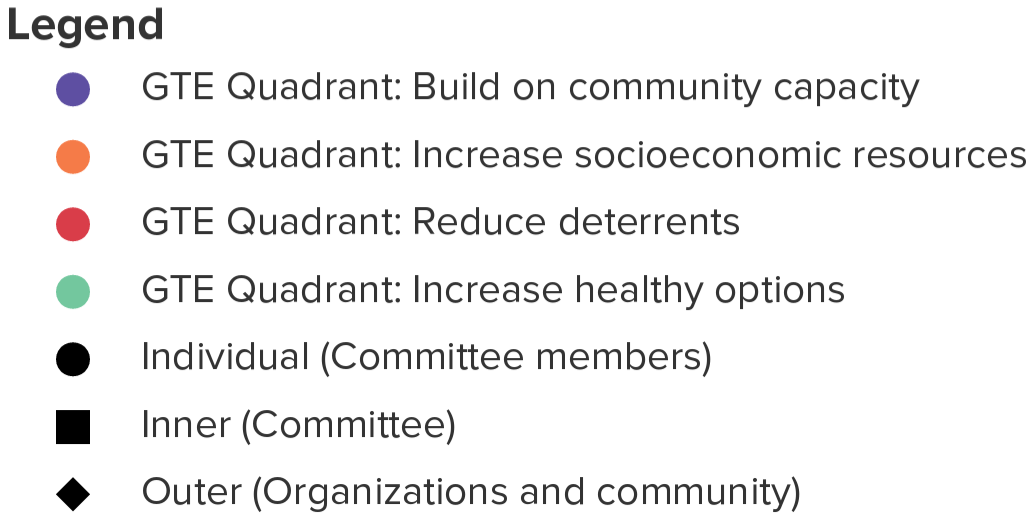

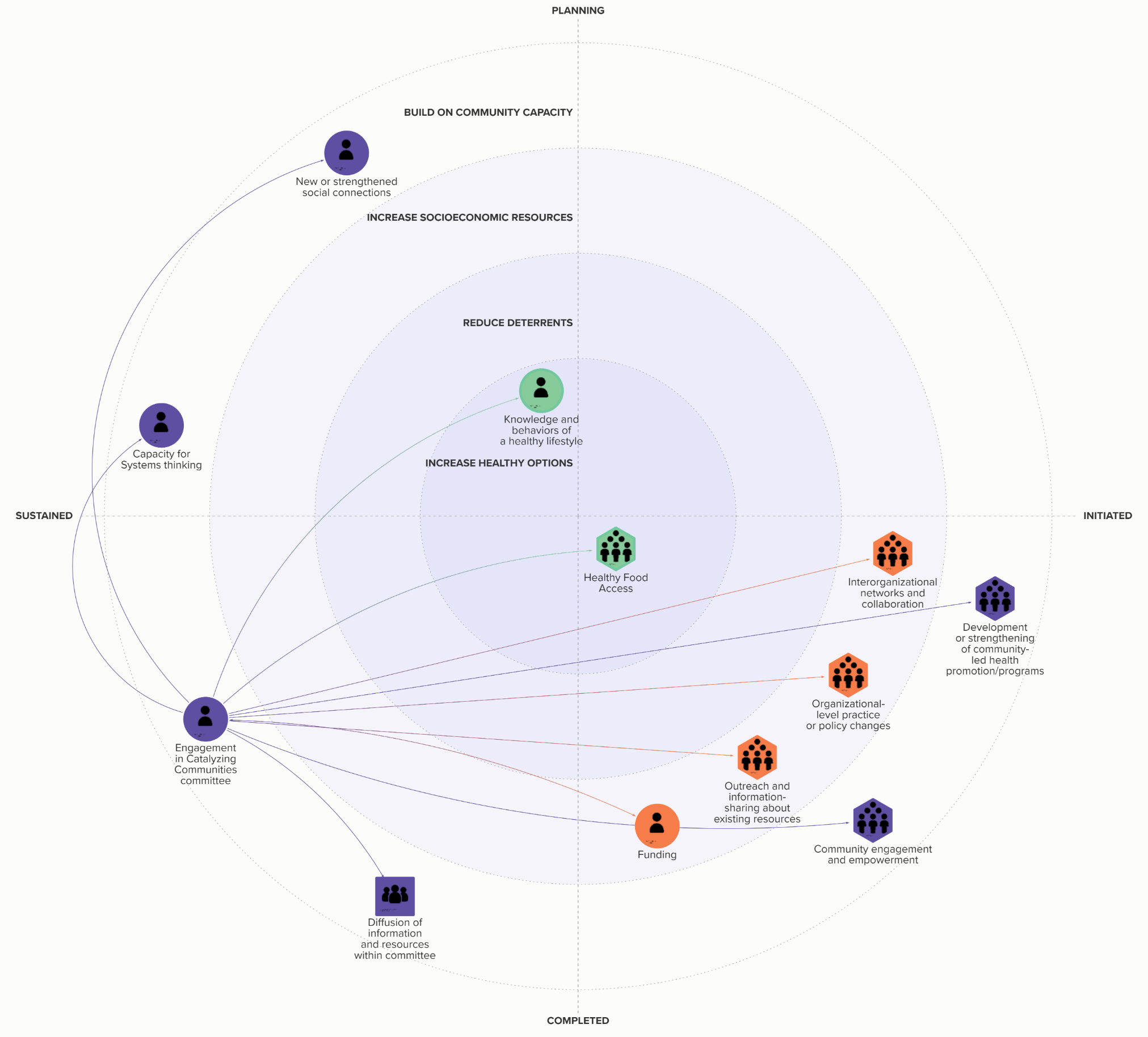

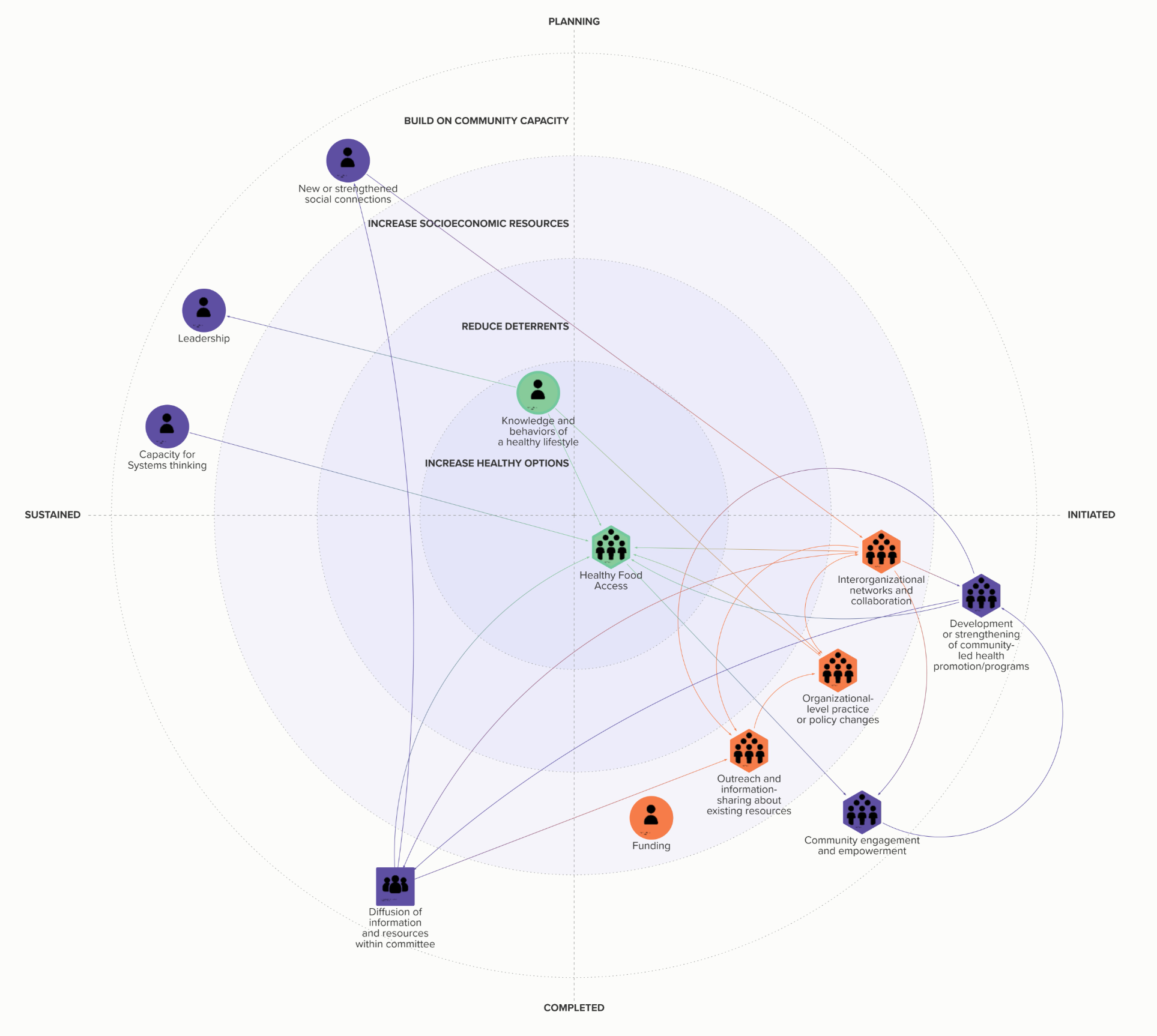

Supplement: Supplementary file 1 — Supplementary file1 (DOCX 3149 kb) [file 11524_2025_1046_MOESM1_ESM.docx]
