# Supplementary material for: Identifying Synergistic Mechanisms of Community-Led Policy, Systems, and Environmental Change for Childhood Obesity Prevention in the Multi-Site Catalyzing Communities Initiative
Source: J Urban Health. 2026 Jan 7;103(1):77–91. doi: 10.1007/s11524-025-01046-y (PMC13136475; doi:10.1007/s11524-025-01046-y)
Supplement: Supplementary file 2 — Supplementary file2 (DOCX 28 kb) [file 11524_2025_1046_MOESM2_ESM.docx]

**Supplementary Table S1.** Codebook

| Code | Definition |
| --- | --- |
| GTE Quadrant 1: Increase Health Options | Barriers, facilitators, or actions to increase healthy options in the community, the specific strategies, and the sustainability of those strategies (Kumanyika, 2019a; Proctor et al., 2011). |
| GTE Quadrant 2: Reduce Deterrents | Barriers, facilitators, or actions to engage the community to reduce deterrents, the specific strategies, and the sustainability of those strategies (Kumanyika, 2019a). |
| GTE Quadrant 3: Increase Social and Economic Resources | Barriers, facilitators, or actions to increase and decrease deterrents for access and availability of social and economic resources, collaborations to achieve this, and sustainability efforts. Also includes statements about efforts to improve social or economic resources available to participants, (e.g., financial assistance or job training programs) (Kumanyika, 2019a). |
| GTE Quadrant 4: Build on Community Capacity | Barriers, facilitators, or actions that strengthen the leadership, social networks, skills, and organizational infrastructure within communities to support long-term health equity goals. Also includes specific actions taken to increase the skills, resources, or organizational capacity of community members (Kumanyika, 2019a). |
| Equity-Focused Synergies | Synergy is defined as the alignment and mutually reinforcing interaction between different interventions, strategies, or policy efforts aimed at reducing health disparities. From a systems perspective, synergy is the emergent and amplified impact that arises from the dynamic, interconnected, and mutually reinforcing interactions between multiple interventions or strategies aimed at reducing health disparities (Wallerstein et al., 2011). |
| Actor Identification | Specific actors (individuals, organizations, or committees) initiating or contributing to actions. This includes changemakers, committee members, local organizations, and other key stakeholders involved in catalyzing change (Wallerstein et al., 2011). |
| Synergy of Resources | Discussions around how synergies specifically helped in addressing health disparities and promoting equity, as seen in the Getting to Equity framework. These synergies refer to descriptions of how participants or organizations combine various types of resources (financial, social, logistical) focused on increasing or reducing access/availability to healthy foods, increasing or reducing access/availability to social or economic resources, or building community capacities, leading to a greater collective impact (Headen et al., 2025; Wallerstein et al., 2011). |
| Collaborative Actions | Instances where participants describe joint efforts across different sectors, organizations, or systems to implement policy, system, and environmental changes (e.g., partnerships between schools and local farms to provide fresh produce) that were focused on increasing or reducing access/availability to healthy foods, increasing or reducing access/availability to social or economic resources, or building community capacities (Lucero et al., 2020; Weiss et al., 2002). |
| Reinforcing Actions/Amplification of Impact | When actions taken in one area (e.g., increasing healthy food access) strengthen or are supported by interventions in another area (e.g., reducing deterrents such as stigma around food assistance programs) (Jackson et al., 2013). |
| Balancing Actions | When actions taken in one area (e.g., increasing healthy food access) hinder or are hampered by interventions in another area (e.g., reducing stigma around food assistance programs) (Jackson et al., 2013). |
| Impacts and Outcomes | Documents the direct and un/observed outcomes resulting from each action, such as changes in behavior, policy adoption, increased access to resources, or improvements in community health indicators (Jackson et al., 2013). |
| Sustainability of Synergies and Impacts | Comments indicating that synergistic efforts have led to more sustainable changes (e.g., lasting policy or system improvements) (Headen et al., 2025; Kumanyika, 2019). |
| Unintended Consequences | Captures outcomes that were not planned or intended, including both positive and negative effects. This could include unexpected social, economic, or environmental impacts that arose as a secondary result of actions (Headen et al., 2025; Jackson et al., 2013). |
| Status of PSE Changes | Indication of progress towards completion of projects/collaborations/formed relationships formed/started/fostered because of the Catalyzing Communities committee. Adapted from (Fawcett et al., 2015). |
| Planning | Planning or just started; one-on-one meetings; forwarding/providing information; planning/strategic meetings; preparing for strategy implementation; collecting and analyzing data; common vision created for mobilization; leadership is singular; no goals have been met; no coalition structure; 1-2 sectors engaged. Adapted from (Fawcett et al., 2015). |
| Initiated or in Progress | Ongoing; bringing/convening more than one stakeholder; enhancing existing services and supports within the community; providing customized TA; providing pre-planned education and/or skill-building sessions typically in group settings; using collected and analyzed data to educate or initiate change; leadership is shared; some goals have been met; formal coalition structure; more than 2 sectors engaged. Adapted from (Fawcett et al., 2015). |
| Completed | Achieved modifying policies, systems, or environments; changing feedback loops, modifying access/opportunities/barriers; leadership is democratic. Adapted from (Fawcett et al., 2015). |
| No Longer in Progress | Strategy has not occurred and/or is no longer in progress. Adapted from (Fawcett et al., 2015). |
| Sustained | How PSE changes are sustained over time, any comments around sustainability of initiatives or enduring relationships/practices/policies. Adapted from (Fawcett et al., 2015). |
| Contextual Factors | Factors unique to a particular setting or individual that contribute to or challenge implementation of PSE changes (Damschroder et al., 2022). |
| Outer Setting (Organization or Community) | Includes the characteristics, dynamics, and culture of committee member organizations. Includes how these characteristics facilitated or hindered increasing healthy options, reducing deterrents, building on community capacity, increasing social and economic resources, and contributing to synergies. Outer setting may include even broader settings beyond their organization, including their coalition, their community (Damschroder et al., 2022). |
| Inner Setting (Committee) | Includes the characteristics, dynamics, and culture of the Catalyzing Communities committee. Includes how these characteristics facilitated or hindered increasing healthy options, reducing deterrents, building on community capacity, increasing social and economic resources, and contributing to synergies (Damschroder et al., 2022). |
| Individuals (Committee Members/Key Actors) | Includes the characteristics of individuals on the Catalyzing Communities committee or key actors identified in the community. Includes how those characteristics facilitated or hindered increasing healthy options, reducing deterrents, building on community capacity, increasing social and economic resources, and contributing to synergies (Damschroder et al., 2022). |
