# Supplementary material for: Identifying Synergistic Mechanisms of Community-Led Policy, Systems, and Environmental Change for Childhood Obesity Prevention in the Multi-Site Catalyzing Communities Initiative
Source: J Urban Health. 2026 Jan 7;103(1):77–91. doi: 10.1007/s11524-025-01046-y (PMC13136475; doi:10.1007/s11524-025-01046-y)
Supplement: Supplementary file 3 — Supplementary file3 (DOCX 17 kb) [file 11524_2025_1046_MOESM3_ESM.docx]

**Supplementary Table S2.** Frequency of impacts (types, statuses, and settings) that emerged through Catalyzing Communities, a Stakeholder-Driven Community Diffusion-informed initiative

| **Impact Categories** | **Impact Sub-Categories** | **Frequencies** | **Examples** |
| --- | --- | --- | --- |
| Type:  Getting To Equity (GTE) Quadrants | GTE 1: Increase healthy options | 72 | - Making healthier selections for food pantries/food giveaway events/community meals - Starting or strengthening community programs that increase access to physical activity opportunities and food - Improving the built environment, such as widening sidewalks for walking or increasing street lighting |
|  | GTE 2: Reduce deterrents | 4 | - Reducing stigma of using food and nutrition assistance programs (e.g., SNAP) or related to child weights - Discouraging neighbors and friends from buying nutritionally low foods |
|  | GTE 3: Improve social and economic resources | 67 | - Collaborations between funding and programming organizations - Sharing information to community residents about local programs that provided social and economic support. |
|  | GTE 4: Build on community capacity | 196 | - Nutrition education that promotes nutrition-focused knowledge and behavior change - Engaging in group model building resulting in causal loop diagrams, a systems-based tool, that promotes systems thinking and deeper understanding of diverse community experiences. - Strengthened interpersonal relationships |
| Status:  Stage of completion | Initiated or in progress | 107 | - Connecting with local representatives and decision-makers towards improving the built environment (e.g., to widen sidewalks for walking) (GTE 1) - Hiring staff to help residents apply for SNAP benefits towards reducing participation stigma (GTE 2) |
|  | Sustained | 66 | - Lasting collaborations with other community organizations (GTE 3) - Ongoing networking with committee members and general feeling of deepened relationships (GTE 4) - Continued use of committee reports to inform organizational activities and funding applications (GTE 3) |
|  | Completed | 54 | - Group model building activities (GTE 4) |
|  | Planning | 48 | - Plans to continue meeting beyond the formal committee structure (GTE 4) |
