# Supplementary material for: Identifying Synergistic Mechanisms of Community-Led Policy, Systems, and Environmental Change for Childhood Obesity Prevention in the Multi-Site Catalyzing Communities Initiative
Source: J Urban Health. 2026 Jan 7;103(1):77–91. doi: 10.1007/s11524-025-01046-y (PMC13136475; doi:10.1007/s11524-025-01046-y)
Supplement: Supplementary file 4 — Supplementary file4 (DOCX 30 kb) [file 11524_2025_1046_MOESM4_ESM.docx]

**Supplementary Table S3.** Synergy themes across GTE quadrants with illustrative and representative quotes

| **GTE quadrant(s)** | **Theme** | **Illustrative quotes** |
| --- | --- | --- |
| 1 and 4 | Systems-thinking capacity strengthened health initiatives | - *By learning the things that I've learned, learning the things that I've learned in the meetings and the diagrams. Just the conversations and listening to the other views of other people, you take away little bits of pieces, "Okay, well, how will that work for us in the things that we're doing?"” (C3)* - *We're showing that we're trying to do healthier choices here because before, we would probably have fried chicken and some macaroni and cheese and corn and cornbread. Those things are good, but we also need to do other things as well. I think that diagram thing really got to me. I really looked at that, and there was so many different things and I was like, "Oh my God." When other groups were saying so many different things, I just learned a whole lot from that diagram. I really enjoyed that.” (C3)* - *I know my supervisor, has definitely mentioned [the causal loop diagrams] or used it in a grant here and there, and just that there's real concrete, not scientific methodology, but evidence, not even evidence-based, but you have a room full of experts. You know what I mean? You're coming out with this, what seems basic, but is actually very concretely, what did we land on, like three areas to focus on? That seems like a good compass. I feel like that's what people, either it's like solidifying what they thought previously as well on like, okay, well, it can't just be a food thing, like plopping a garden in a low-income neighborhood is not going to be the solve-all, and things like that, which I think is always really important, especially for a food justice organization to remember that it is not just about the food. It's about lots of other things that are going on around it.” (C1)* |
| 1 and 4 | Advocacy for structural changes to expand healthy options. | - *Being more active in other committees that are advocating for street widening with sidewalks, advocating for what's coming into our area, speaking up when they're talking about the grocery store and how come we don't have one. Maybe not immediate, but hopefully eventually. It's helping me make slow impacts here and there.” (C2)* - *“What do we need?" "What is needed to move things forward to be able to know, "Okay, is there an area where there needs to be a city presence?" I think I'm the only one from the city in that area.” (C1)* - *For me, it's become a more intentional conversation at work in all the different ways that this can come up. We just talked about involving young people, and systems change, and laws.” (C1)* |
| 1 and 4 | Enhanced capacity to promote healthy lifestyles | - *We're starting walking groups based out of the [community health center]. They have a nice little park and lake. We already do it for our patients, and we're looking now to expand it and bring in the actual community too to say, "Hey, at least once a month, come out and join us. We're going for walks. You have the ability to talk with a healthcare provider if you have any questions." That's the biggest things that we've started doing to face [health] issues.(C2)* - *I would say my mom walking group was [an action that stemmed from this committee], talking about obesity, talking about our health, how we feel. It's a change of lifestyle as we age. I think those are big conversations.” (C1)* - *[Connecting with families and getting their opinions on things has been connected to the work of the committee] because it's nutrition, it's educating [families] as well because a lot of them don't know how much fruits and vegetables the kids should have, and a lot of them is like, "Okay, so I don't give them this, he doesn't like that," and it's opening the doors for them to try new things with the children, samples, that's what I always call them. Sample everything, and even if you don't like it, sample it anyway" (C3)* |
| 1 and 4 | Increased awareness and shifts in professional approach to strengthen community health | - *I think it's brought attention to eating habits, to physical activity. With the older residents, I think the nutrition part has been enlightening, and for the younger groups, I think the physical activity has really gotten them involved. There was a time many years ago where we would have a turkey trot around Thanksgiving. For some reason, it just went by the wayside and I guess more than 10 years, it didn't happen. One of the committee members is involved with a physical activity program in the district and he instituted it again. I'm really glad that that's happened because families can come out and participate. (C2)* - *I think the efforts that this project has done is good because I think it's opened the eyes of a lot of people because members of this committee are somewhat leaders of the community and communicate with people. I think the feeling is there that we need to find ways to be able to offer opportunities to learn about good health, good cooking and exercise, and creating family activities that the whole family can participate in. Maybe it's a walk for a purpose of some type, or either walking clubs.” (C2)* - *One of the things that we are slightly changing on the food insecurity is we're adding culturally relevant foods. That wasn't included, so we're trying to get more programs that are supporting the culturally relevant foods. We've built pantries in schools within [the community], and when we stock the pantry, we get the basic staples, the spaghetti, the ravioli, stuff like that. We haven't thought about the Spanish foods and other nationalities. Whenever we try to do it now, whenever we do stock the pantry now, we're adding stuff like the black beans and the rice and soy sauce and stuff like to support the other ethnicities. [...] Because this was not an all-white committee, this was a committee made up of different ethnicities, so I could learn more about what their needs are.” (C1)* - *What we do here on our parent meetings since I've been a part of the community, we push nutrition more. During our once-a-month parent meeting, we talk about nutrition, we do little workshops with the parents. When the kids do their food experience in the classroom. We would normally do stuff that's healthy, but we do healthy, healthy now. (C3)* |
| 1 and 4 | Community events as catalyst for engagement and capacity building | - *It seems each time we have a meal, we're reaching different individuals and it is I want to say diverse, so we're having the different races come out. [The meals are] centered around Black culture wellness but we have other individuals who are coming who are in the community who want to know what's happening in their community as well and who want to have a seat and have these conversations and give their input. I think that that's a wonderful thing because we're all neighbors and we're all in the community together. I think that's what the committee and the meals and stuff that the action plan that we decided upon, how that brought that change and it's growing from there. Having someone else come in and helping us with the meals and seeing that it's important so they're going to help and pick up with funding, and to keep it going and to make it more intentional, and to really reach and gather data, and all of that. It's grown from just we're going to get together and have a meal and talk about things. We're talking about them, and hopefully making change as well through these conversations.” (C3)* - *[Regarding impact that participating in this committee has had on the community as a whole] I think the new girl that's taking over the group, she's very involved, and that's the location that starts all these physical activities, the parties getting involved, involving the community and different things.” (C2)* |
| 1 and 3 | Awareness of and access to community health programs through social networks | - *Talking to different people like [committee member’s name redacted]. She has opportunities for children to swim. She's connected with [a university] and [a non-profit organization that offers physical activity programming]. [...] How do we get the word out? Organizations that are funded need community support to get the word out. If you're offering this, you should have a A, B, C, D, E plan, a plan to implement it, and also to outreach the community.” (C1)* - *The committee has had influence because I know our boss was really adamant about getting this study in [our community]. Just knowing that he wanted to make sure that he was aware of what was really going on, and make sure that we address the main issues of how we can assist this community [...]. We've provided, a small portable clinic[...] We have a mental health pod there as well, so we're definitely making changes to change the way that they think about health in [community], and this committee has definitely helped because we bring whatever information we learn from here, we bring it on to make sure that we're making those changes for them. Now, the participation of us in the committee, an organization, well, now that we have implemented so many changes, it's not only affecting the community, we also have other people from all around the [city] come and take advantage of our programming [...]. It wouldn't have happened as fast, I don't think, if we weren't aware of what was really going on with what the stakeholders have brought to the table and the knowledge that they have given us. (C2)* - *A lot of my co-workers, my peers have said they sent parents to [programs I learned about through the committee]. They gave the information as far as the bus taking them to the grocery store. They have used that information. I've had parents that let me know that they use the bus service to go. They are using the information that we give them to participate. I know my peers are giving that to their parents as well. (C3)* |
| 1 and 3 | Strategic funding applications to advance health programs | - *[Participating in this committee] I've realized that it's wonderful to have the passion, but if you don't understand the system you cannot fulfill it. Being in this committee, it helped me really understand the full nuances and detail of leveling, of breakdown, of understanding that you have to be able to communicate in order to get the funding. [Funding applications], that would probably be very overwhelming and challenging to have to respond in a way that makes you think deeper or see it on those extra levels. I had to go through this [committee] to understand how to frame my program in a way that it could be fundable, like take it outside of passion, and I had to put it on paper program-wise. I had to break down, "Well, what are the goals? Who is it serving? Why? How are we serving them? How does this relate?" Then through going through this, it helped me to start breaking my program down. (C1)* - *One of the things we talk a lot about being a community foundation is that we are just stewards [of money] at a moment in time. It's not our money, it's the community's money, and the importance of listening to the community. I think that community has answers. How do we infuse that into our work a little bit more to [issue funding applications]. Looking at the makeup of the committee, how can we replicate some of that to be having more intersectional conversations, and looking at how we can do our work differently. (C3)* |
| 1 and 2 | Reframing physical activity to reduce weight stigma | - *We had registration for our classes and there was a family who came and they purchased the membership, the family membership, and it was a mom and a dad and two kids. The mom told me straight up, she's like, "The reason we purchased the membership was because the doctor said that my daughter is obese." Her daughter was seven years old. When she said that in front of the child, the child was just looking down. It broke my heart because I don't feel like that's something she should say in front of the child. The problem was there was conflict with her age. [...] Normally before this committee, I would have been like, "I'm so sorry. She needs to wait," but of course, I've learned a lot since being in this committee. It broke my heart. I've reflected a lot on my childhood and looking at her, it broke my heart. I made an exception and I let the child in the class. Of course, I told the little girl before they left, her name was [name redacted], and I said, "[name redacted], you are beautiful just the way you are. [...] This class, don't go there to lose weight, go to have fun." When I told her this, she just lit up. (C2)* - *The girls [program]. They have their open gym time where we'll do basketball and stuff. However, it's for girls by girls. I have a girls fitness trainer. During their gym time, she'll come in and do exercising with them, take them through actual group exercises. The goal is during those times to be talking about like body image, self image, because like the social media puts so much pressure. It's crazy. Nobody's good enough. (C1)* |
| 2 and 4 | Enhanced systems-thinking capacity to identify barriers | - *I think the big barrier that I will call out, I would say is just getting everyone on the same page. Calling attention to "This is important, this is an issue." "Why?" you'd think. There's certain things you would think would be common knowledge, but it's not, or there's just not an understanding or there are different priorities and agendas going on, or whatever it might be. I would say [presenting the causal loop diagrams has] been in general conversations. I'm looking forward to being able to, at some point, have that, again, more public knowledge. I'm sure that's on the radar for other folks. Again, it's just in my mind. We haven't spoken on it either, but in terms of education and combating stigma, I feel like it would be really helpful. (C1)* - *We're also trying to find partners. Partners for childcare. While we don't have the means to pay for this childcare, are there service providers that would be willing to show up to meetings so that if people can participate, and this is again, assuming it's at a time where someone's off work, that if we're asking you to participate, we are lowering the barriers to your participation.” (C3)* - *I don't see nothing wrong with the dynamics that they do when they draw everything on the board because you get a better vision when you're sitting there looking at it, more so, because now you're sitting right there in front of the board, it. I'm that type of person. I do have to see it. My field is about gun violence and domestic, but we starting to open the doors for it and the childhood obesity, it's slowly coming in with this too. (C3)* |
| 3 and 4 | Social capital to strengthen community capacity | - *The committee's out there doing things. We've got different partnerships within the committees doing different projects so it's not just one thing that we're doing. Each subcommittee is doing their own thing to address it from all different angles. (C2)* - *I would say my approach is, and it falls into my previous answer, my approach is more a collaborative approach. I's not a one-man show. I can't do it by myself.” I think one of my key changes in how I do my work is to collaborate. If I don't know, it's okay to ask an outside organization. I think this is one thing that we actually came up with in the committee that it's not a contest, it's not a competition. I have gotten further with collaborating and bringing in those key stakeholders so that we're giving our communities a fighting chance. (C3)* - *You're seeing a higher enthusiasm around getting people information about public, lots more public benefits, and a lot of the same groups are talking to each other. I think that that doesn't get old. I think it's important that there's redundancy in that. We're all reminding each other of different sectors that are coalescing together and that, again, reinforcing the fact that they all play together. (C1)* - *When I see [the committee] now, we bonded a lot during these meetings and it was just so diverse, the group, in terms of not ethnically necessarily, but in terms of sectors. It was just so diverse that it just pretty much expanded the network that I have in different fields. (C1)* |
