# Supplementary material for: Identifying Synergistic Mechanisms of Community-Led Policy, Systems, and Environmental Change for Childhood Obesity Prevention in the Multi-Site Catalyzing Communities Initiative
Source: J Urban Health. 2026 Jan 7;103(1):77–91. doi: 10.1007/s11524-025-01046-y (PMC13136475; doi:10.1007/s11524-025-01046-y)
Supplement: Supplementary file 5 — Supplementary file5 (DOCX 21 kb) [file 11524_2025_1046_MOESM5_ESM.docx]

**Supplementary Table S4.** Contextual factors across individual, inner, and outer settings that reinforced or counteracted action implementation or impacts towards PSE (policies, systems, or environmental) changes with illustrative and representative quotes

| **Setting** | **Theme** | **Type of synergy** | **Illustrative quotes** |
| --- | --- | --- | --- |
| Individual | Prior participation in other committees, taskforces, or working groups | Reinforcing | - *As a representative of the school district, I serve as a school board member, I have shared along the way with the other trustees what the committee has been doing (C2)* - *I gather information and I take that and I go out and I share that with other committees that have an impact. I'm talking to other people that our county engineers, city engineers and I'm giving them information like, […] I'm having discussions with people that are from the community and this is where we're at. I'm taking what I have here and I'm going out and expressing it to people that I know are going to help me make that change or help me get to where we need to be. Maybe not immediate, but hopefully eventually. It's helping me make slow impacts here and there. (C2)* - *What we do here on our parent meetings, we push nutrition more. During our once-a-month parent meeting, we talk about nutrition, we do little workshops with the parents. When the kids do their food experience in the classroom, we would normally do stuff that's healthy, but we do healthy, healthy now. They'll do like tossed salads, fruit salads and things like that. Because I noticed here where I'm at, we have a lot of kids when we do their nutrition assessments, that [have] obes[ity]. (C3)* - *[I’m involved in] a food task force. Well, I go when I can go, but I was in there and I had mentioned that they need to have a food pantry. I live near [street name redacted]. They don't have no food pantry. I had brought my concern up to [committee member’s name redacted] and he was like, "Oh, I didn't know that. I thought there was a food bank in [...] area." He looked it up. He was like, "You're right, there's no food bank. I'm going to take that in and do some homework on it." I heard yesterday through a parent that they're talking about putting a food bank in our area. (C1)* |
| Individual | Leadership skills | Reinforcing | - *I have a full entire afterschool program basically that I'm about to start running that includes an audio visual program that's going to help the kids either create a podcast or a show of their own. We've got the cooking program, which is going to focus on cultural equity, but then those kids from the audio video program are going to film and record the kids for the recipe program so that we can put it out to the public and say, "Hey, public, this is the cultural recipe we're doing for this week. Make it for your homes but, hey, package and label these and go put them in one of the five fridges. That's what I'm doing with my program […] it's been like 40 hours of my life or more, it's like a full-time job, but when you care about it, you do it. (C1)* - *I think the new girl that's taking over the group, [committee member’s name redacted], she's very involved, and that's the location that starts all these physical activities, the parties getting involved, involving the community and different things. I think it's going to work out good because she's up there and she's got all the pulls and all the buttons to press, so it's probably going to be a success. If not, it's going to be a good start.” (C2)* - *It's like you know how you have a little fire, right? You like this little fire like a candle. It's a little fire. It feels good. It's warm. It makes sense. That's where I was as far as nutrition and my discussions with my clients at first. As a result of being around other people on the team and talking to other people on the stakeholder committee, I'm like, "No, this needs to be a forest fire. This is a bigger conversation that needs to happen. This is way more important than, especially for my community, because we have a higher occurrence of high blood pressure and diabetes, heart problems. It's a very necessary conversation." (C3)* |
| Inner | Committee facilitation | Reinforcing | - *The young lady on the team, I think her name is [facilitator’s name redacted]. I think she's absolutely dynamic; to see such a young person so involved in the community in such a huge way with her heart and her actions. (C3)* - *[Facilitator’s name redacted] is just amazing. She kept it all together and she keeps you focused and she draws from you and she makes you feel important in whatever little tidbit you have. She could brighten it up for everybody. It was really great. (C2)* |
| Inner | Cross-organizational resource exchange within the committee | Reinforcing | - *This was the first time I actually worked with a group that had the same vision and mission and goals and outline, but from different perspectives, which was really cool. With that, we were able to network. This is when we realized it's better to work as a team with external organizations than trying to do it all on your own. We're duplicating work, we might as well work together to really get to the community the way that we need to. (C3)* - *We're all reminding each other of different sectors that are coalescing together and that, again, reinforcing the fact that they all play together. (C1)* |
| Outer | Tangible and financial support | Reinforcing | - *I think that the committee and the meals and the action plan that we decided upon, that brought change and it's growing from there. Having someone else come in and help us with the meals and seeing that it's important so they're going to help and pick up with funding, and to keep it going and to make it more intentional, and to really reach and gather data, and all of that. It's grown from just we're going to get together and have a meal and talk about things. (C3)* - *While we want to have people's participation, we also need to be thoughtful about compensating for that. (C3)* - *It's not easy to just bring programming, it takes a lot of funding. (C2)* |
| Outer | Pre-existing programs, organizational capacity, and partnerships | Reinforcing | - *It was just validation. The validation that you get when you are part of something that it's constantly sharing, like up-to-date best practices. When you come back to your college or wherever you work, your agency, or wherever you're representing, and you say, hey, I'm part of this, these are some ideas that they have, it's got more traction, it just creates more validation on whether that is a good idea or not. Adopting those are definitely easier sell. (C1)* - *One thing I've been making sure to do in our monthly meetings that we have for our civic association is to include resources that I obtained from [other committee members]. That focus on childhood health and well-being. (C2)* - *Organizationally, we're so big, so [participating in the committee] probably just made like a ripple, but I think it's the smallest impacts may ultimately lead to greater impacts in the community. There has always been that push to have work, health and wellness in the organization. I think with, with this Committee, it's like move it now, like there is urgency in getting things accomplished and relationships and events going. (C3)* |
| Outer | Local-level policies | Reinforcing | - *I think the school district and our government, our leadership, need to get together and work together to [promote physical activity]. (C2)* - *It'd be great to push back on [the local housing authority] a little-- actually, a lot, because they are accountable. I think they should be responsible and engaged and care about whether the children living in the spaces that they maintain are accessing these spaces, and they're not. They have the power to do that. They're doing a lot of work. […] The Coalition has a connection to [the local housing authority], but it's politics. I think a group like this [committee] could be really instrumental in pushing that. (C1)* |
| Outer | Community unsafety | Balancing | - *If your community is not safe, you don't want to get out and walk […] People don't want to go out walking, or get out and socialize. (C2)* - *We're living in poverty still. With the violence comes on a lot of numerous things. People be really just scared to even go outside. They might get what they going to get from [the grocery store] and hurry up and get right back in the house. The violence has plagued our community so bad to where people be scared to even come outside to go to the grocery store. (C3)* |
| Outer | Stigma | Balancing | - *I still have not been able to convince [my daughter] but I got a membership to [gym] and I wanted her to go with me, and she's like, "No Mom I am not going with you to the gym. I don't need to go to the gym." I'm doing that. I'm trying to talk to her because I want her to see that she doesn't have to be obese to exercise. (C2)* - *There's still a lot of stigma to food banks. I know better because I did food distribution, so I'm like, "You got to know where to get it." Not every family sees it the same way. There's a lot of pride involved. (C1)* |
| Outer | Low affordability of food and physical activity opportunities | Balancing | - *There was an initiative here that was pretty good in the sense of being creative with regards to accessibility of healthy foods like the farmers' markets that were happening in areas that were populated by a lot of the people that can not necessarily afford these organic foods or these better foods for them. (C1)* - *A lot of parents still can't eat the way I eat because everything is so expensive, because they have kids that they're raising versus me, it's just me and my husband. It is really making things affordable in the community to the people that we serve on a day-to-day basis. (C3)* - *We have [health centers that promote physical activity], but the parents can't afford to go. (C3)* |
| Outer | Commercial interests | Balancing | - *You go to [fast food restaurant], a salad is $8, a cheeseburger or a hamburger is $0.99. I don't think that they really want us to be healthy. Keeping us unhealthy keeps money in their pockets [...] I don't think they're related to promoting [child health equity]. I think they're related to reducing. I think those are direct impacts which are not being addressed, which cause health to be inequitable. (C1)* - *In this area, all you see is fast food locations. [...] Do you see a grocery store? No, we don't. We don't have that. That definitely contributes to child obesity. Instead of the families or parents driving a little bit farther to go to the grocery store to get healthy produce. It’s always easier, let me just go right here down the street to get some [fast food restaurant]. That is contributing to child obesity, for sure. (C2)* |
